# Incommensurately modulated structure of Zn_4_Si_2_O_7_(OH)_2_·H_2_O at high pressure

**DOI:** 10.1107/S2052252524011060

**Published:** 2025-01-01

**Authors:** Roman Gajda, Wojciech Sławiński, Tomasz Poręba, Jan Parafiniuk, Mohamed Mezouar, Przemysław Dera, Krzysztof Woźniak

**Affiliations:** a Faculty of Chemistry University of Warsaw Pasteura 1 Warszawa 02-089 Poland; b European Synchrotron Radiation Facility 71 Avenue des Martyrs Grenoble 38000 France; c Institute of Geochemistry, Mineralogy and Petrology, Department of Geology University of Warsaw Żwirki i Wigury 101 Warszawa 02-089 Poland; d Hawaii Institute of Geophysics and Planetology, School of Ocean and Earth Science and Technology University of Hawaii at Manoa 1680 East West Road, POST Bldg, Office 819E Honolulu Hawaii96822 USA; e Department of Chemistry, Biological and Chemical Research Centre University of Warsaw Żwirki i Wigury 101 Warszawa 02-089 Poland; ESRF, France

**Keywords:** high-pressure phase transitions, modulation, incommensurate structures, hemimorphite, diamond anvil cells

## Abstract

High-resolution single-crystal X-ray diffraction experiments on Zn_4_Si_2_O_7_(OH)_2_·H_2_O hemimorphite were conducted at high pressure using diamond anvil cells at several different synchrotron facilities (ESRF, Elettra, DESY). Experimental data confirmed the existence of a previously reported phase transition and revealed the exact nature of the incommensurate modulation. We report the incommensurately modulated structure described in the (3+1)D space group *Pnn*2(0, β, 0)000. We have determined the modulation mechanism, which involves the fluctuation of atoms between two main positions, occurring mainly along the [100] direction, perpendicular to the modulation vector. Moreover, our results reveal that the phase transition occurs at lower pressure than previously reported.

## Abstract 2

Refinement of the hemimorphite crystal structure using single-crystal synchrotron X-ray diffraction data collected at high pressure revealed a structural phase transition into an incommensurately modulated structure, accompanied by the appearance of satellite reflections.

## 1. Introduction

**Figure 1:**
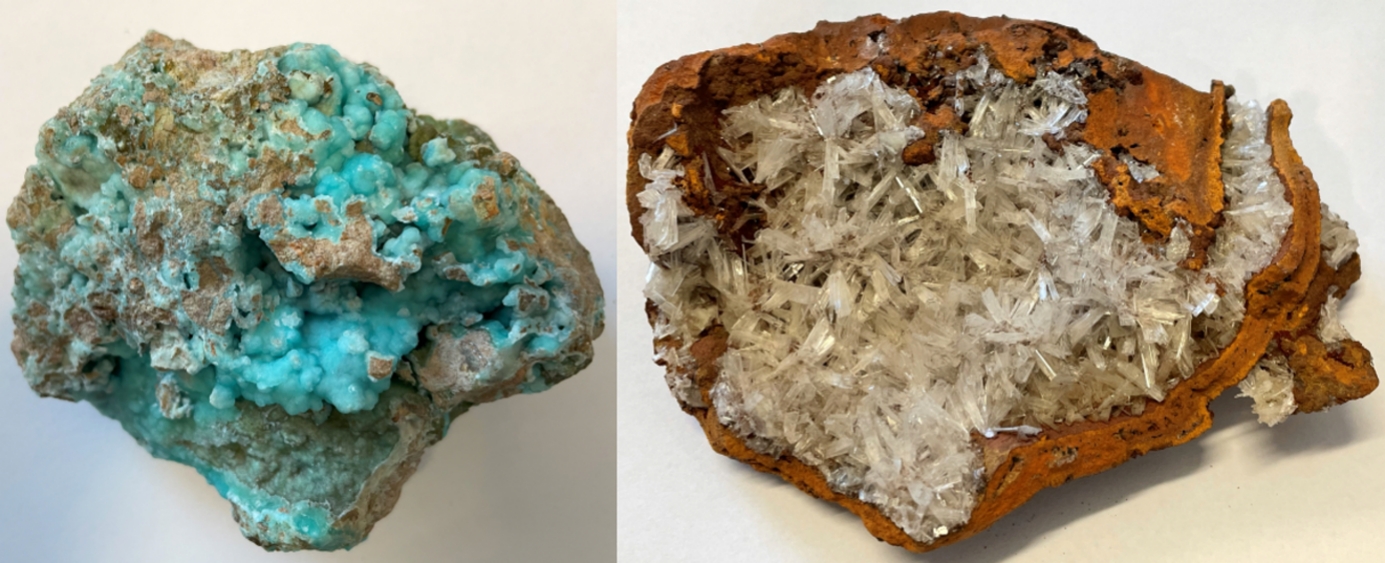
Specimens of hemimorphite: (left) bluish crystals contain an admixture of copper and (right) colourless crystals without significant admixtures.

Hemimorphite [**Hmp** – symbol approved by the International Mineralogical Association, Commission on New Minerals, Nomenclature and Classification (Warr, 2021 ▸)] is one of the most common minerals in non-sulfide Zn deposits. In 1853 Adolph Kenngott gave the name in allusion to the hemimorphic morphology of the crystal derived from the Greek words hemi (‘half’) and morph (‘shape’) because each end of an **Hmp** crystal has a different shape. Many names were previously assigned to these species, including calamine, which is still sometimes encountered, but hemimorphite was chosen by the International Mineralogical Association, over calamine, in 1962. The mineral often forms very well developed tabular or short prismatic crystals, colourless or white. Botryoidal, stalactitic and mammillary blue masses are also known (see Fig. 1 ▸).

‘Non-sulfides’ is a term which comprises a series of oxidized Zn(Pb)-ore minerals (Boni & Mondillo, 2015 ▸). **Hmp** forms from the oxidation products of sphalerite and other zinc minerals, especially in arid climates. It is common in the oxidized and supergene zones of hydro­thermal deposits, where it is associated with smithsonite. The supergene deposits are formed by weathering and oxidation at ambient temperatures. The mineral is economically important and is mined in many locations around the world as a component of oxidized zinc ores. An anhydrous variety of zinc silicate is Zn_2_SiO_4_ willemite, which crystallizes in a trigonal structure with tetrahedrally coordinated Zn and Si (Klaska *et al.*, 1978 ▸). Willemite is known to undergo as many as four high-pressure phase transitions on compression to 15 GPa (Marumo & Syono, 1971 ▸). Most recently, **Hmp** studies have been focused on phenomena such as its elastic properties or pyroelectricity (Li & Bass, 2020 ▸; Wu *et al.*, 2023 ▸). **Hmp** is also interesting in that it could be considered a biomineral. Fourier transform infrared spectroscopy (FTIR) and X-ray absorption spectroscopy (XAS) analysis have revealed that an extracellular product of biomineralization conducted by cyano­bacterium *Leptolingbya frigida* is a crystalline phase closely resembling **Hmp** (Medas *et al.*, 2018 ▸).

The first record in the Inorganic Crystal Structure Database (ICSD; Belsky *et al.*, 2002 ▸) of the X-ray structure of **Hmp** refers to a paper published in 1932. In this work, the unit-cell dimensions, space group and atomic positions were determined for the first time (Ito & West, 1932 ▸). In later studies during the 1960s, the geometry of the SiO_4_ tetrahedra was re-examined and redetermined (Barclay & Cox, 1960 ▸; McDonald & Cruickshank, 1967 ▸). In the 1970s, **Hmp** was studied for the first time with the use of neutron diffraction and the hydrogen-bonded system was investigated (Hill *et al.*, 1977 ▸; Takéuchi *et al.*, 1978 ▸).

**Figure 2:**
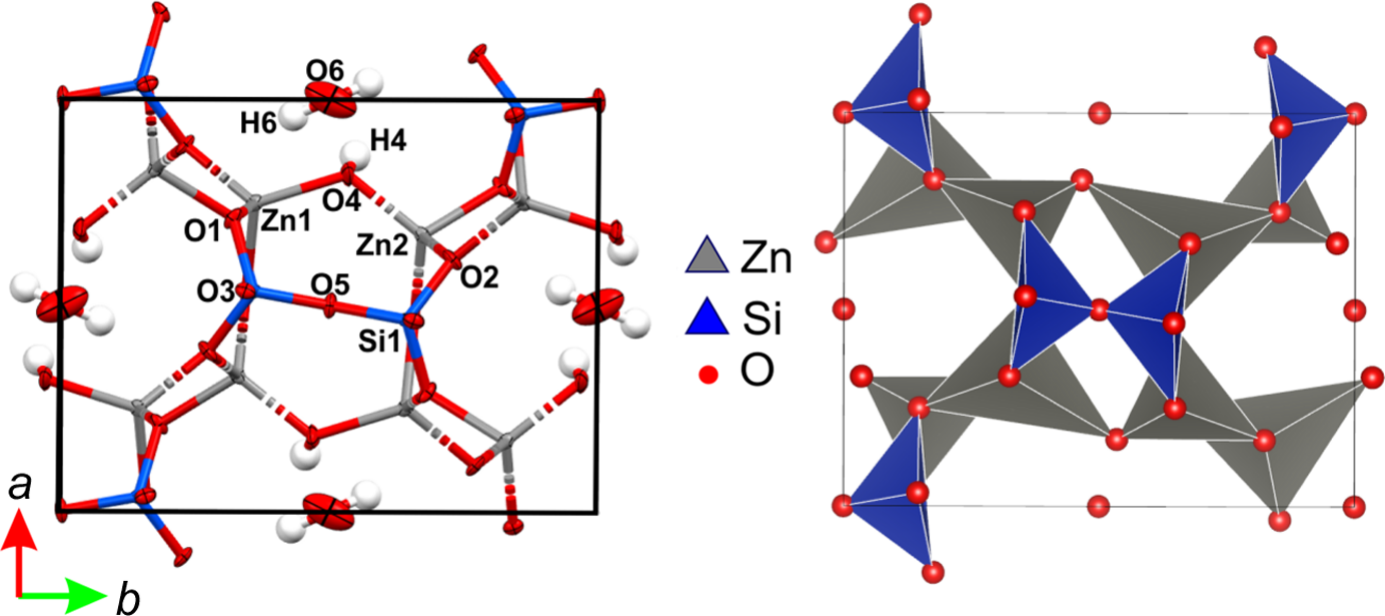
Unit cell of **Hmp**. ADPs and labels of atoms (left) and in the polyhedral representation (hydrogen atoms omitted for clarity).

The crystal structure of **Hmp** is relatively simple. Under normal conditions (RT and ambient pressure) it crystallizes in the space group *Imm*2 (see Fig. 2 ▸). The basic building blocks are SiO_4_ and ZnO_3_OH tetrahedra (Fig. 2 ▸). The oxygen atom of the hydroxyl group is shared between two neighbouring Zn tetrahedra. The water molecule is located in a structural cavity and locked via four hydrogen bonds with four surrounding hydroxyl groups. The mineral formula is Zn_4_Si_2_O_10_H_4_ and there are two molecules within the unit cell (*Z* = 2).

The mineral was also investigated under non-ambient temperature and pressure. When the sample is heated to 600°C (Cooper *et al.*, 1981 ▸), a contraction of the structure caused by dehydration is observed. Proton disorder in the dehydrated structure of **Hmp** was also investigated (Libowitzky *et al.*, 1997 ▸).

When crystals were cooled to 20 K, a phase transition from *Imm*2 to *Abm*2 was found (Libowitzky *et al.*, 1997 ▸, 1998 ▸). This particular phase transition is characterized by a doubling of the *c* parameter. In our research, we have already observed this phase transition at 110 K. It is accompanied by the appearance of superstructure satellite reflections. The modulated structure after the phase transition is commensurate, **q** = (0, 0, 0.5), which is why it is possible to solve the structure in the unit cell with doubled lattice parameters and a conventional space group.

High-pressure investigations of **Hmp** within the pressure range between ambient pressure and 4.2 GPa revealed that the phase transition occurs between 2.44 and 3.17 GPa (Seryotkin & Bakakin, 2011 ▸). Incommensurate satellite reflections along **q** = (0, 0.119, 0) were reported, and the average structure of the high-pressure phase was refined at 3.01 GPa in the space group *Pnn*2, ignoring the modulation (Okamoto *et al.*, 2021 ▸).

Interestingly, the mineral bertrandite, Be_4_Si_2_O_7_(OH)_2_ (space group *Cmc*2_1_), which is topologically identical to **Hmp**, was investigated under pressure up to 4.1 GPa and does not show any phase transition within this pressure range (Hazen & Au, 1986 ▸).

Current knowledge about **Hmp** includes that at room temperature and ambient pressure it occurs in the ortho­rhombic space group *Imm*2. The low-temperature phase transition leads to a transformation into another ortho­rhombic space group *Abm*2 with a doubled *c* lattice parameter [or equally described as a commensurate modulation with modulation vector **q** = (0, 0, 0.5)], whereas a high-pressure phase transition leads to the space group *Pnn*2 [incommensurate modulation, **q** = (0, 0.119, 0) (Okamoto *et al.*, 2021 ▸)]. All the transitions between space groups such as *Imm*2 → *Abm*2 or *Imm*2 → *Pnn*2 are caused by rearrangements of SiO_4_ and ZnO_4_ polyhedra and/or subtle changes of hydrogen bonds towards the water molecules. The main goal of this study was to investigate the mechanism of the high-pressure phase transition.

## 2. Results

### 2.1. Experimental

Our experiments were conducted, above all, at the ID27 beamline at the European Synchrotron Radiation Facility (ESRF) dedicated to high-pressure measurements (Poręba *et al.*, 2022 ▸). For our data collection, we used λ = 0.2229 Å and a small 2 × 2 µm beam. The wavelength has been calibrated using a CeO_2_ (*e.g.* NIST SRM 674b) powder standard. Single-crystal data were collected using an Eiger2 X 9M CdTe detector through ±32° ω scans and a step size of 0.5° around one rotation axis. After transformation into the Esperanto format, the *CrysAlisPro* program suite was used for indexing and data reduction (Rigaku, 2014 ▸). Corrections for X-ray absorption effects [by the diamond anvil cell (DAC) components] were applied using the semi-empirical ABSPACK routine implemented in *CrysAlisPro*. The structures were refined with *ShelXL*, as incorporated in *Olex2*. Three single crystals of **Hmp** were selected and loaded into a membrane DAC in which helium gas was used as the pressure medium. The experiment was conducted at pressure values between ambient and 4.1 GPa (the pressure was determined by measuring the position of the fluorescence lines of ruby; pressure uncertainty: 0.03 GPa). The main goal was to observe the consequences of a phase transition reported to occur somewhere between 2.5 and 3.0 GPa (Seryotkin & Bakakin, 2011 ▸). Datasets were collected at ten pressure values, eight below the possible phase transition and two above. At each pressure point, data were collected separately for each of the three single crystals in the DAC. Additionally, at each pressure point, each single-crystal dataset was measured with three different combinations of experimental parameter such as exposure time and slit width. As a result, over 90 individual datasets were collected. The reason for conducting the measurements in this way was that access to the reciprocal space of single crystals in a DAC is significantly restricted. Individual datasets collected as a step scan with rotation about one axis have relatively low completeness, even when the orientation of the crystals in the DAC is optimized. To achieve reliable completeness, merging of datasets is necessary. Also, to reduce the number of unmeasured reflections and oversaturated reflection intensities, it is worth using different parameter settings for data collection.

In addition to measurements conducted at ID27 at ESRF, we also collected X-ray data at other beamlines/synchrotrons, such as the Xpress beamline at the Elettra synchrotron facility at Trieste and the beamline P24 at Petra III Deutsches Elektronen-Synchrotron (DESY) in Hamburg.

On the beamline P24 at Petra III (DESY, Germany), three different single crystals were tested separately in different DACs (Merrill and Bassett design). X-ray diffraction measurements (λ = 0.35424 Å) were performed under pressure on the beamline equipped with a PILATUS 1M CdTe detector. The measurement strategy was a combination of phi scans from −36 to +36° on a four-circle kappa diffractometer (EH1). The whole strategy consisted of runs with different exposure times (2.0, 1.0 or 0.5 s) and different frame widths (1 or 0.5°). For crystal 1 we used pressures of 2.94, 3.26, 3.5 and 3.8 GPa; for crystal 2 we used pressures of 2.49, 3.05 and 3.11 GPa; and for crystal 3 we used pressures of 1.92, 2.8, 3.19 and 4.1 GPa.

The results obtained for data collected at ESRF were also confirmed at another synchrotron facility, on the Xpress beamline at Elettra in Trieste, Italy. The X-ray diffraction measurements (λ = 0.4956 Å) were performed under pressure with a *ca* 50 µm beam on the beamline equipped with a PILATUS3 S 6M (DECTRIS). The detector was placed *ca* 226 mm from the sample crystal. The diffraction data were collected using phi scans from −38 to +38° and a step size of 0.5° around one rotation axis. We used a pressure of 2.57 GPa (the pressure medium was 1-propanol).

Data reduction was performed using the *CrysAlisPRO* software (Rigaku, 2014 ▸). The structure was solved and refined with *ShelXS* (Sheldrick, 2008 ▸) and *ShelXL* (Sheldrick, 2015 ▸), respectively, within the *Olex2* suite (Dolomanov *et al.*, 2009 ▸). Attempts to solve and refine modulation were undertaken in the *Jana2020* software (Petricek *et al.*, 2023 ▸).

### 2.2. Phase transition

Incommensurately modulated (IC) phases occur when the atomic structure of a mineral adopts a periodic modulation that does not align with the underlying lattice periodicity. In nature, it is fairly uncommon to find minerals which have strong and sharp incommensurate Bragg reflections. However, several good examples exist, including natrite, calaverite, melilite, fresnoite, pearceite, polybasite and cylindrite which confirm long-term stability of incommensurate phases (Bindi & Chapuis, 2017 ▸). Incommensurate modulation can arise or disappear as a result of changes in thermodynamic conditions. High pressure can induce distortions in the crystal lattice, causing atoms to shift from their ideal positions, leading to periodic modulations that are not commensurate with the original lattice periodicity. Different vibrational modes of the lattice can also couple in complex ways under high pressure, creating new periodicities that are incommensurate with the original lattice. Ca_2_MgSi_2_O_7_ akermanite transforms from an IC phase, stable at room pressure, to a commensurate phase at 1.33 GPa (Yang *et al.*, 1997 ▸). Brownmillerite-type Ca_2_Al_2_O_5_ transforms to an incommensurately modulated structure above 1000 K (Lazic *et al.*, 2008 ▸). Low-temperature commensurate ferrimagnetic α-Mn_3_O_4_ undergoes a commensurate–incommensurate magnetic transition at 33 K (Kemei *et al.*, 2014 ▸). Incommensurate modulation of atomic positions can also be coupled with magnetic moments as already demonstrated for inorganic CaMn_7_O_12_ (Sławiński *et al.*, 2009 ▸), where a structural phase transition into incommensurate modulation occurs below 250 K and also below 90 K Mn magnetic moments form a helical spin arrangement.

The incommensurate modulation can be an energy-minimizing configuration under high pressure, as the complex arrangement can lower the free energy of the system compared with a commensurate structure. High pressure can also enhance competing interactions within the crystal, such as between different types of bonding or between different sublattices, leading to a compromise structure that is incommensurate. Anharmonic effects, or non­linearities in the potential energy surface, become more significant at high pressure and can stabilize incommensurate modulations. Changes in electronic structure under high pressure can drive the formation of incommensurate phases, as pressure can alter the distribution of electronic density, leading to new bonding patterns that are incommensurate. Atoms may experience displacive modulations, where they are displaced periodically from their average positions, creating an incommensurate periodicity. Another common type of modulation is occupancy modulation, where two atom types sharing the same crystallographic position modulates its occupancy.

On the basis of data retrieved from the ICSD, the values of the unit-cell parameters for the *Imm*2 **Hmp** under ambient conditions vary as follows: *a*: 8.191(1)–8.388(1) Å, *b*: 10.714(1)–10.824(2) Å and *c*: 5.088(1)–5.115(3) Å (Ito & West, 1932 ▸; Barclay & Cox, 1960 ▸; McDonald & Cruickshank, 1967 ▸; Hill *et al.*, 1977 ▸; Takéuchi *et al.*, 1978 ▸; Cooper *et al.*, 1981 ▸; Libowitzky *et al.*, 1997 ▸). A likely source of the unit-cell variation are admixtures present in natural minerals. Therefore, after averaging, the unit-cell dimensions are as follows: *a* = 8.289 Å, *b* = 10.769 Å and *c* = 5.102 Å. Shrinking of the unit-cell dimensions as a function of pressure is quite subtle. As we know from the literature (Seryotkin & Bakakin, 2011 ▸), the unit-cell dimensions under 2.44 GPa (RT, *Imm*2) are *a* = 8.2209(9) Å, *b* = 10.6920(15) Å and *c* = 5.0614(2) Å. Note that some of the earlier reports used natural samples exhibiting some ion substitution [*e.g.* the sample studied by Okamoto *et al.* (2021 ▸) had about 1.6 wt.% of P].

**Figure 3:**
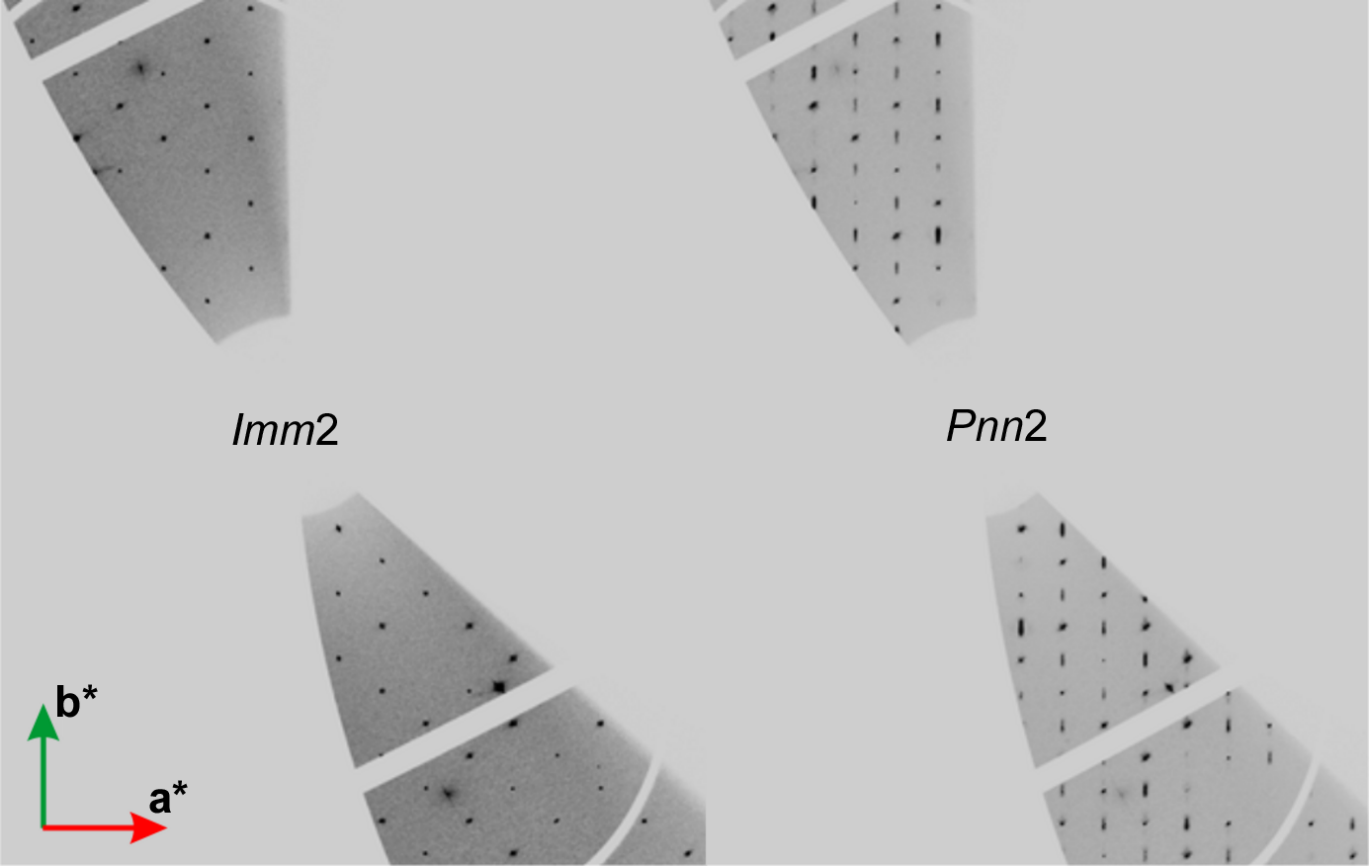
Comparison of the *hk*0 reciprocal lattice layers for the structure of **Hmp** in the space groups *Imm*2 (left; 1.0 GPa) and *Pnn*2 (right: 3.3 GPa). Additional reflections that were forbidden in *Imm*2 are observed in *Pnm*2. Data collected on beamline ID27, ESRF.

The first high-pressure studies of **Hmp** (Seryotkin & Bakakin, 2011 ▸) revealed that the phase transition from *Imm*2 to *Pnn*2 occurs between 2.44 and 3.17 GPa, but no incommensurate modulation was reported. The accompanying changes of the unit-cell parameters are relatively subtle. The *a* parameter changes by less than 1%, *b* by about 1% and *c* by about 0.3% [difference between 2.44 and 3.17 GPa (Seryotkin & Bakakin, 2011 ▸)]. Fortunately, the change of the space group and the proof of a phase transition can be clearly verified by comparing diffraction patterns of specific crystallographic layers. In the case of *Imm*2, reflections must fulfil the following condition, within the group of *hk*0 reflections: *h* + *k* = 2*n*. In the case of *Pnn*2, this condition is no longer valid. As a result, it is sufficient to check *hk*0 layers if *h* + *k* = 2*n* + 1 reflections are observed or not (see Fig. 3 ▸). Although the unit-cell dimensions are almost identical, a simple comparison of the *hk*0 diffraction patterns tells us if a phase transition has already occurred or not, which is quite visible in our data collected at ESRF (see Fig. 3 ▸).

**Figure 4:**
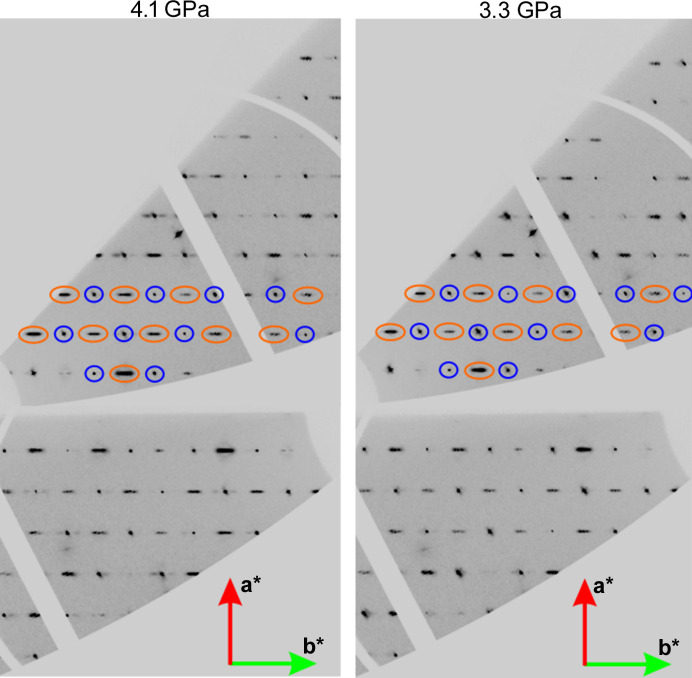
Combined reciprocal layers of the *hk*0 reflections for the pressure points 3.3 and 4.1 GPa measured for **Hmp**. Selected reflections already visible for the space group *Imm*2 are marked in navy blue. Reflections which appear as a consequence of the phase transition to the space group *Pnn*2 are marked in orange.

During the data collection at ESRF, we measured four pressure points in the vicinity of the previously reported transition pressure: 2.3, 2.6, 3.3 and 4.1 GPa. The reciprocal lattice layers of the *hk*0 reflections determined for two pressure points, 3.3 and 4.1 GPa, are presented in Fig. 4 ▸. Both are well above the phase transition. In addition to peaks marked with blue circles, which were already observed in the *Imm*2 space group, new peaks marked with orange circles appeared, indicating the new *Pnn*2 symmetry.

The additional reflections in the *Pnn*2 space group appear broader. This is a consequence of the fact that accompanying satellite reflections have a relatively short modulation vector. These satellite reflection appear as a result of the incommensurate modulation. The modulation vector, which describes the modulation direction and position of satellite reflections, is relatively short (the b* component varies between 0.105 and 0.179), which makes three reflections (one Bragg peak and two satellites) appear as one streak. In the high-pressure phase, the satellite reflections are visible along the [010] direction.

In the case of the low-temperature *Imm*2 → *Abm*2 phase transition, the modulation vector was rational (equal to 0.5 b*), therefore it indicated commensurate modulation (a supercell). As a consequence, it was possible to index the data, solve and refine the structure by simply using the doubled lattice parameter along the direction where the satellite reflections appeared. In the case of the high-pressure phase, the modulation vector varies between 0.105 b* and 0.179 b* (depending on the pressure). That is why the same motive could be repeated after between five and nine unit cells, depending on the precise value of the modulation vector.

IC modulated structures cannot be approximated by 3D periodic structures. This is because the atoms are periodically modulated according to a modulation function with a period that is incommensurate to the periodicity of the crystal lattice. Therefore, the real structure of **Hmp** is not periodic in 3D but can be described as periodic in (3+1)D space. The displacive type of modulation occurs when atoms deviate from their basic structure positions.

**Figure 5:**
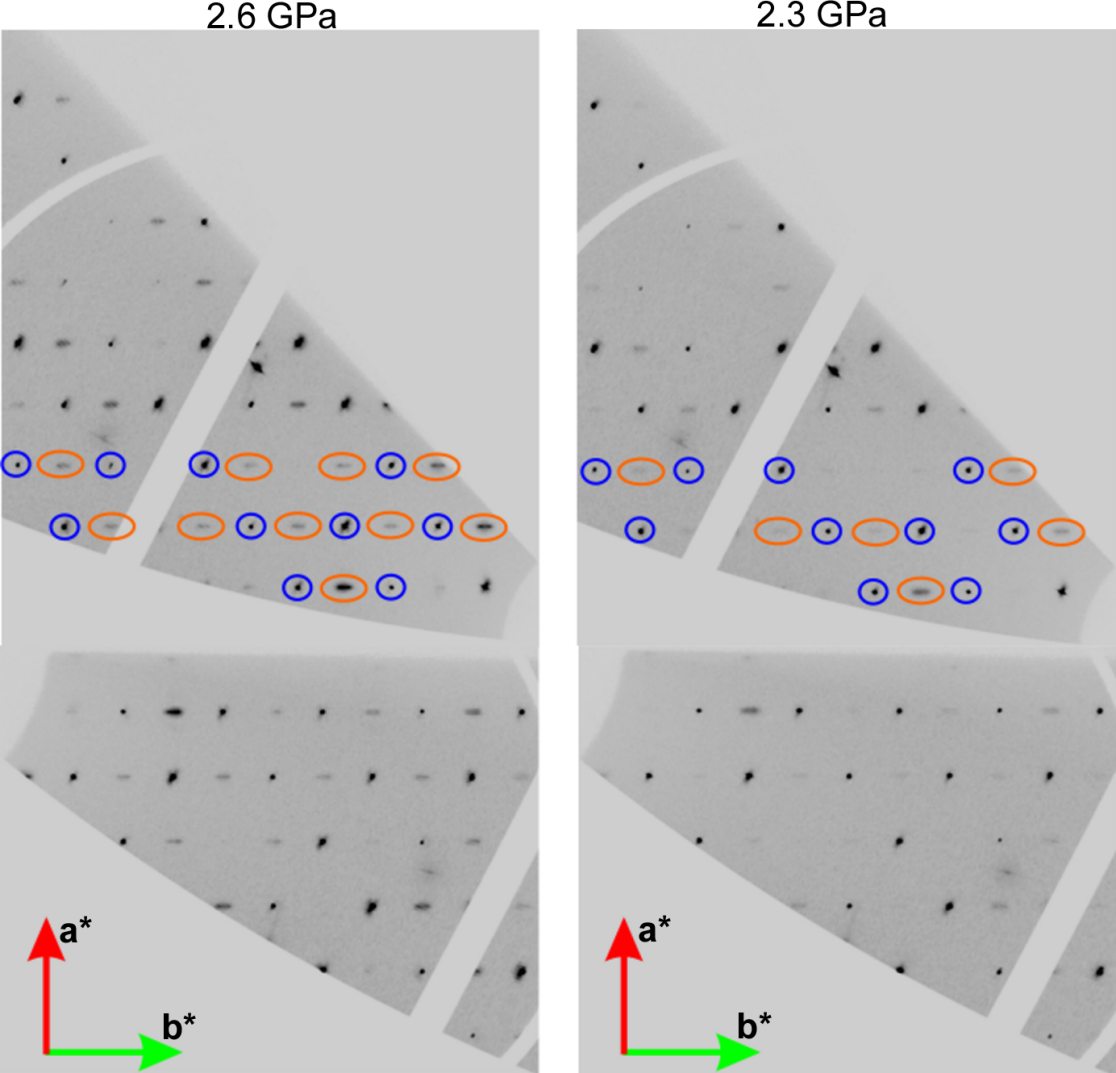
Combined reciprocal layers of *hk*0-type reflections for pressure points 2.6 and 2.3 GPa. Within navy blue boundaries the selected reflections are already visible in the space group *Imm*2. Within orange boundaries the reflections appear as a consequence of the phase transition to the space group *Pnn*2.

Even more interesting are two other pressure points, 2.6 and 2.3 GPa. This is because, so far, only the structures of **Hmp** above 3 GPa were refined as structures after the phase transition from *Imm*2 to *Pnn*2. However, in our measurements, the layers of *hk*0 reflections definitely confirm the existence of the broad diffused reflections which do not fulfil the *h* + *k* = 2*n* rule and are accompanied by satellites. These broad reflections prove that, at 2.6 GPa, the phase transition has already occurred and the structure should be solved and refined in the space group *Pnn*2 (see Fig. 5 ▸). The same is also true for 2.3 GPa, where additional reflections and satellites are much weaker but do also exist. At the onset of the phase transition the reflections and their satellites have relatively low intensity and could be easily overlooked.

**Figure 6:**
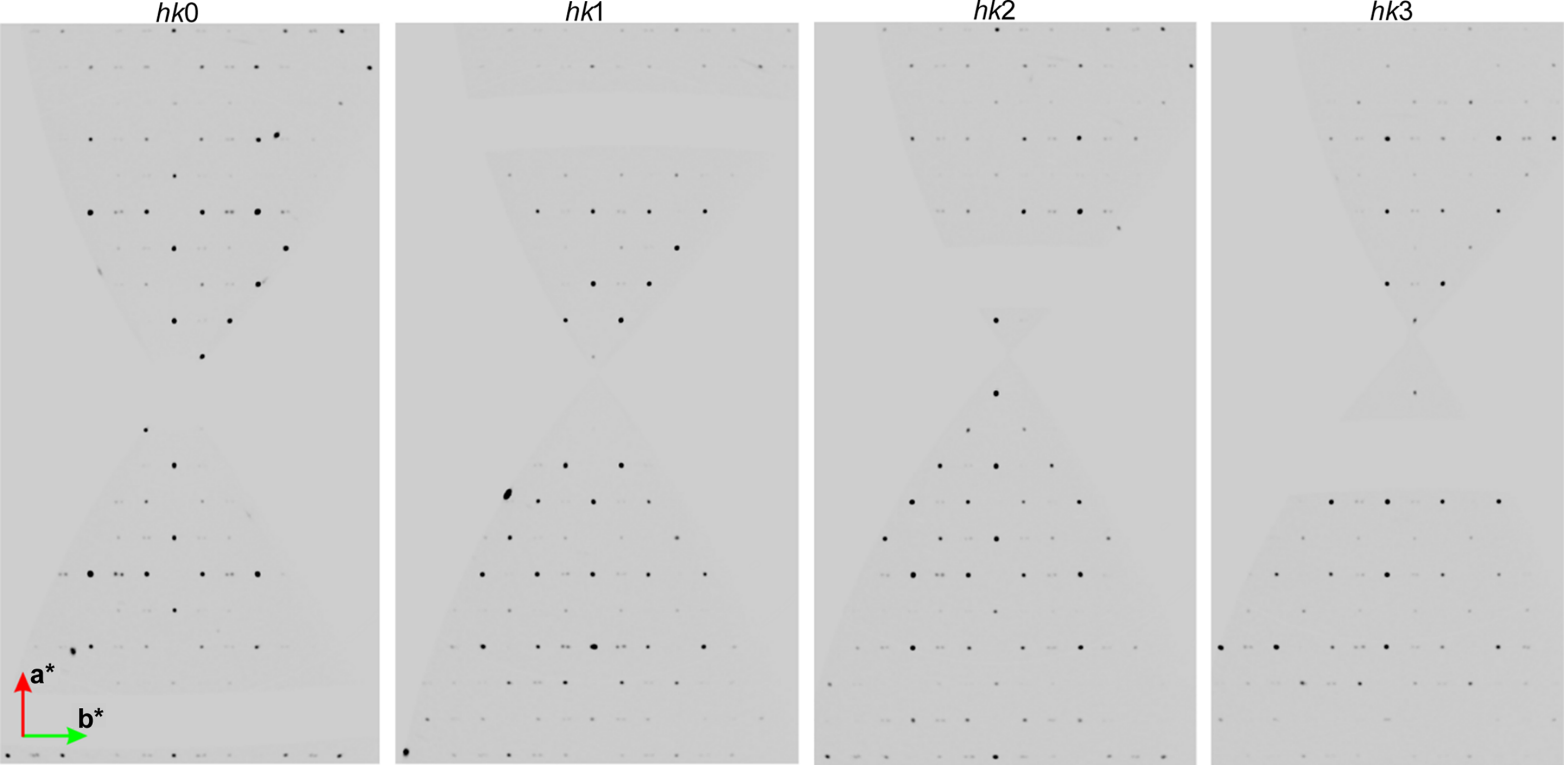
Reciprocal lattice layers of reflections *hk*0, *hk*1, *hk*2 and *hk*3 for **Hmp** under 2.8 GPa pressure – P24 dataset (Petra III, DESY).

For the datasets collected on beamline P24 at Petra III (DESY, Germany), the satellite reflections were observed only in one case, for crystal 3 at 2.8 GPa (the pressure medium was 1-propanol; see Fig. 6 ▸). The quality of the datasets collected was lower than in the case of ID27 (ESRF).

**Figure 7:**
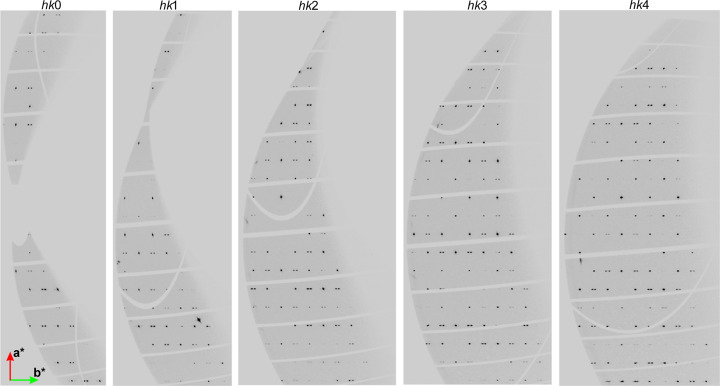
Reciprocal lattice layers of the reflections *hk*0, *hk*1, *hk*2, *hk*3 and *hk*4 for **Hmp** under 2.57 GPa pressure – Xpress dataset (Elettra).

The satellite reflections from the modulated structure after the phase transition, collected on the Xpress beamline at Elettra in Trieste, Italy, are visible in Fig. 7 ▸. Unfortunately, the datasets collected on Xpress have too-poor completeness to be used to solve and refine the structure.

#### 2.2.1. Comparison of results from different beamlines

At the core of this paper are results from measurements on ID27 at ESRF. This is due to the fact that datasets collected on this particular station present the best quality. Because we were looking for satellite reflections, by ‘best quality’ we mean the fact that satellite reflections were observed and they were relatively strong and data completeness was relatively high. The satellite reflections accompanying the high-pressure phase *Pnn*2 appear very close to the main Bragg’s reflections (the value of the modulation vector is small). That is why to detect them a stable and intense beam is required as well as a sensitive detector. Each beamline has different hardware characteristics. The wavelengths of the X-ray beams applied during measurements conducted at ESRF (ID27), Petra III (P24) and Elettra (Xpress) were as follows: 0.2229, 0.3542 and 0.4956 Å, respectively. In the case of ID27 and Xpress, the size of the beam spot was small enough to measure separately 2–3 single crystals closed in a DAC. This is convenient when one wants to merge *hkl* datasets. In the case of high-pressure experiments with DACs, access to reciprocal space is restricted, hence the type of scans on a particular goniometer that are possible is important. The best option from the point of view of completeness is when the measurement strategy can be combined from phi and omega scans and different detector positions. It seems that at least in the case of **Hmp** and hunting for satellite reflections with a small **q** value, the most important factor was the wavelength of the beam.

**Figure 8:**
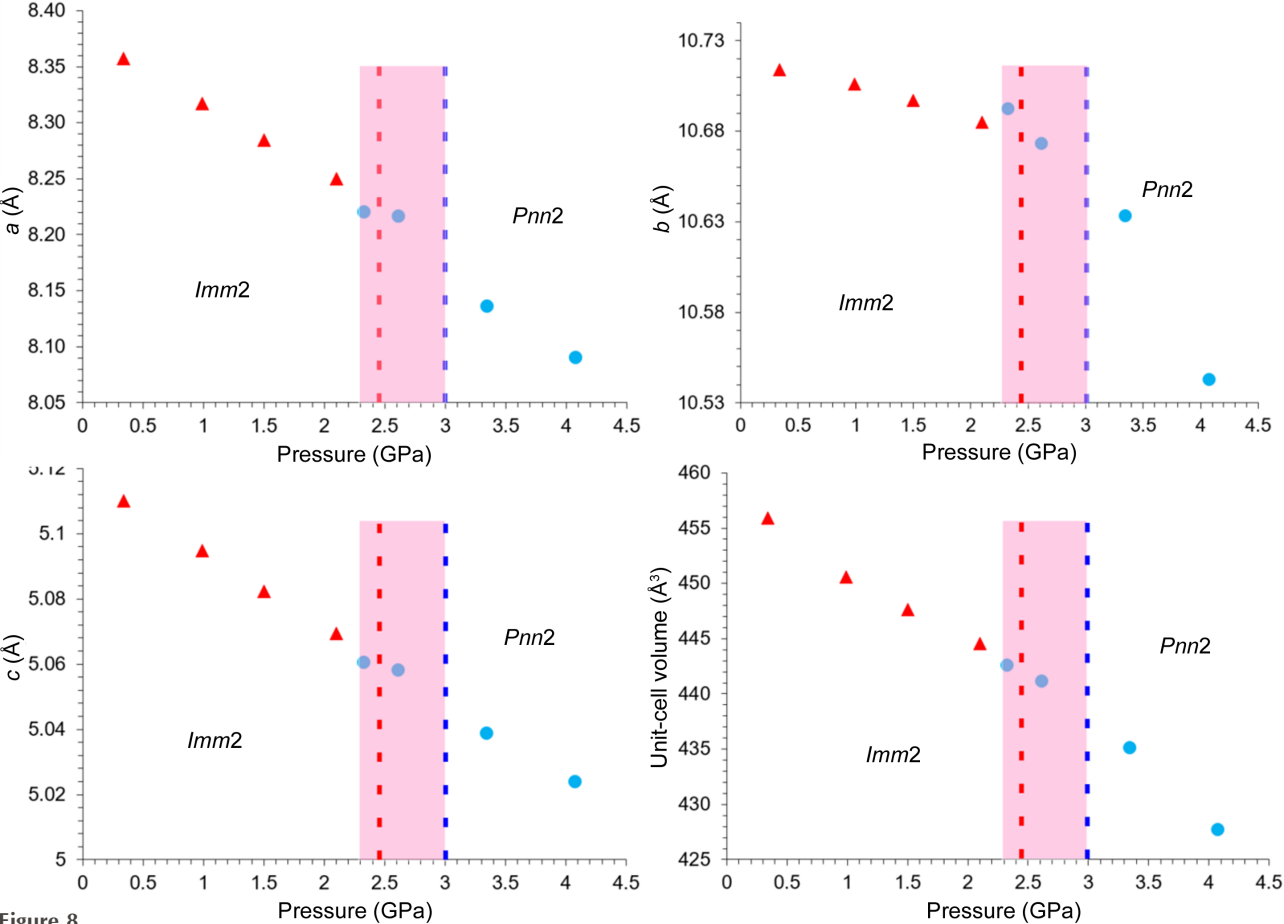
Unit cells and cell volumes as a function of pressure. Description of the symbols in the main text.

The changes of the unit cells and cell volume of **Hmp** as a function of pressure are shown in Fig. 8 ▸, where the results collected at ID27 are presented. The charts also indicate where the phase transition from *Imm*2 to *Pnn*2 occurs. The red dotted line (*p* = 2.44 GPa) indicates the highest pressure under which the *Imm*2 phase was determined by Seryotkin & Bakakin (2011 ▸). The blue dotted line (*p* = 3.01 GPa) indicates the lowest pressure under which, as known from the literature, the *Pnn*2 phase was determined by Okamoto *et al.* (2021 ▸). The area between these two lines was considered as the pressure range within which the phase transition should occur. However, because we now know that the phase transition from *Imm*2 to *Pnn*2 probably occurs slightly earlier, we extended this range, depicted as the pink region. The pressure points where the structure was solved in the space group *Imm*2 (before the phase transition) are depicted as red triangles and the pressure points where structure was solved in the space group *Pnn*2 (after the phase transition) are depicted as blue circles.

Taking into account measurements in the vicinity of this range, we observe satellites under 2.3 and 2.6 GPa (ID27 ESRF), 2.58 GPa (Xpress, Elettra), and 2.8 GPa (P24, Petra III). This observation confirms that the phase transition occurs below 3.01 GPa (to the left of the blue line) or even below 2.44 GPa (to the left of the red line). There are two datasets, both collected on P24 at DESY, which were collected at pressures of 2.49 and 2.94 GPa for which we do not observe satellite reflections.

#### 2.2.2. Average structure refinement

Our experiments confirmed our observation of the earlier reported phase transition and the IC modulated nature of the high-pressure phase (Seryotkin & Bakakin, 2011 ▸). After the phase transition, the extent of modulation increases with increasing pressure, making it harder and harder to ignore in structure determination. Earlier reports included average structure refinements, ignoring the modulation, but this simplification resulted in artefacts such as unusual bond lengths and anomalously large and anisotropic atomic displacement parameters. To properly account for modulation, the structure has to be solved in the (3+1)D space group consistent with the indexing of the satellite reflections and modulation vector **q** [either *Pnn*2(0, β, 0)000 or *Pnn*2(0, β, 0)*s*00]. Parameters characterizing our results at the level of the independent atom model refinement (on the basis of *F*^2^) of the average structure (ignoring modulation) are shown in Table 1 ▸. In the case of datasets collected at 2.6 GPa, it was possible to merge data from two separate single crystals. This was done after data reduction in *CrysAlisPro* using the so-called ‘profit merge’ function.

After the phase transition, individual crystals in the DAC showed slightly different magnitudes of the modulation vector, which made merging of the datasets impossible. In the case of one of the crystals, the associated dataset shows satellite reflections of the second order but completeness of this separate measurement is only *ca* 50%. In the case of the refinement of the average structure, the effect of modulation increases at higher pressure, as shown by increasing refinement figures of merit. Just above the phase transition (2.6 GPa), the average structure approximation looks acceptable (peaks in residual density maps are relatively low). At higher pressures, when atomic movements are more significant, higher *Q* peaks are observed in residual density maps next to the main average atomic positions. This is a strong indication that point modulation should be taken into account. Because of these significant *Q* peaks, obtaining satisfactory atomic displacement parameters for selected atoms is no longer possible, which is why, at 4.1 GPa, two oxygen atoms were refined with only isotropic displacement parameters.

Table 2 ▸ presents the bond lengths and angles that describe the geometry of the average structures of **Hmp**. The table consists of the results from this study (datasets collected at ID27) as well as from the literature.

### 2.3. Hydrogen bonds in the structure of hemimorphite

There are two hydrogen atoms in the asymmetric part of the structure of **Hmp**. One of them belongs to the water molecule, which is placed on the twofold axis, and the second one is part of a hydroxyl group and connected with an oxygen atom that bridges two zinc atoms. However, in the high-pressure experiments, it is not always possible to locate both of these hydrogen atoms due to insufficient quality of the data (low completeness). Among the 22 structures currently deposited in the ICSD, there are more than ten structures without water hydrogens and eight structures without any hydrogen position determined. In our studies, we were able to find and refine positions of the hydrogen atoms for data collected at 2.6 GPa. In this case, the modulation vectors of two single crystals were comparable and it was possible to merge datasets and obtain higher completeness. At higher pressures, due to differences in modulation vector values, data merging was not possible and we refined the structures of the high-pressure phase without hydrogen atoms. The geometry of hydrogen bond type O(4)—H(4)⋯O(4) which forms at 2.6 GPa is as follows: *d*(O—H) = 1.051 Å, *d*(H⋯O) = 1.999 Å, ∠*D*HO = 164.28°, *d*(O⋯O) = 3.024 Å.

### 2.4. Modulation vectors

In our experiment, the individual single-crystal samples at the same pressure showed different lengths of the modulation vector. As a result, it was not possible to merge data from several crystals to increase completeness. Crystals measured at 2.57 GPa in silicone oil at the Xpress beamline at Elettra give **q** = 0.105 b*. Crystals measured at 2.6 GPa in He at the ID27 at ESRF give **q** = 0.09 b* (in this case it was possible to merge data from two separate crystals, see Table 1 ▸). The discrepancy between the lengths of the modulation vectors increased with pressure, suggesting that the effect of nonhydro­static and deviatoric stress was responsible for the effect. For three single crystals measured simultaneously in one DAC under 3.3 GPa (ID27, ESRF), the lengths of the modulation vectors are 0.166, 0.104 and 0.148, respectively. At 4.1 GPa the modulation vectors for those crystals are as follows: 0.179, 0.107 and 0.152. Because the satellite reflections for this third crystal under 4.1 GPa were the most significant (second-order satellite reflections were observed), this particular measurement served as a data source for the refinement in *Jana2020*. On the basis of information from Professor Leonid Dubrovinsky and Natalia Dubrovinskaia (private communication), such a phenomenon that particular single crystals supply different values of the modulation vectors can be treated as a new type of polymorphism, which was also found in their studies of other crystals under pressure (Yin *et al.*, 2024 ▸).

### 2.5. Refinement of the modulated structure

**Figure 9:**
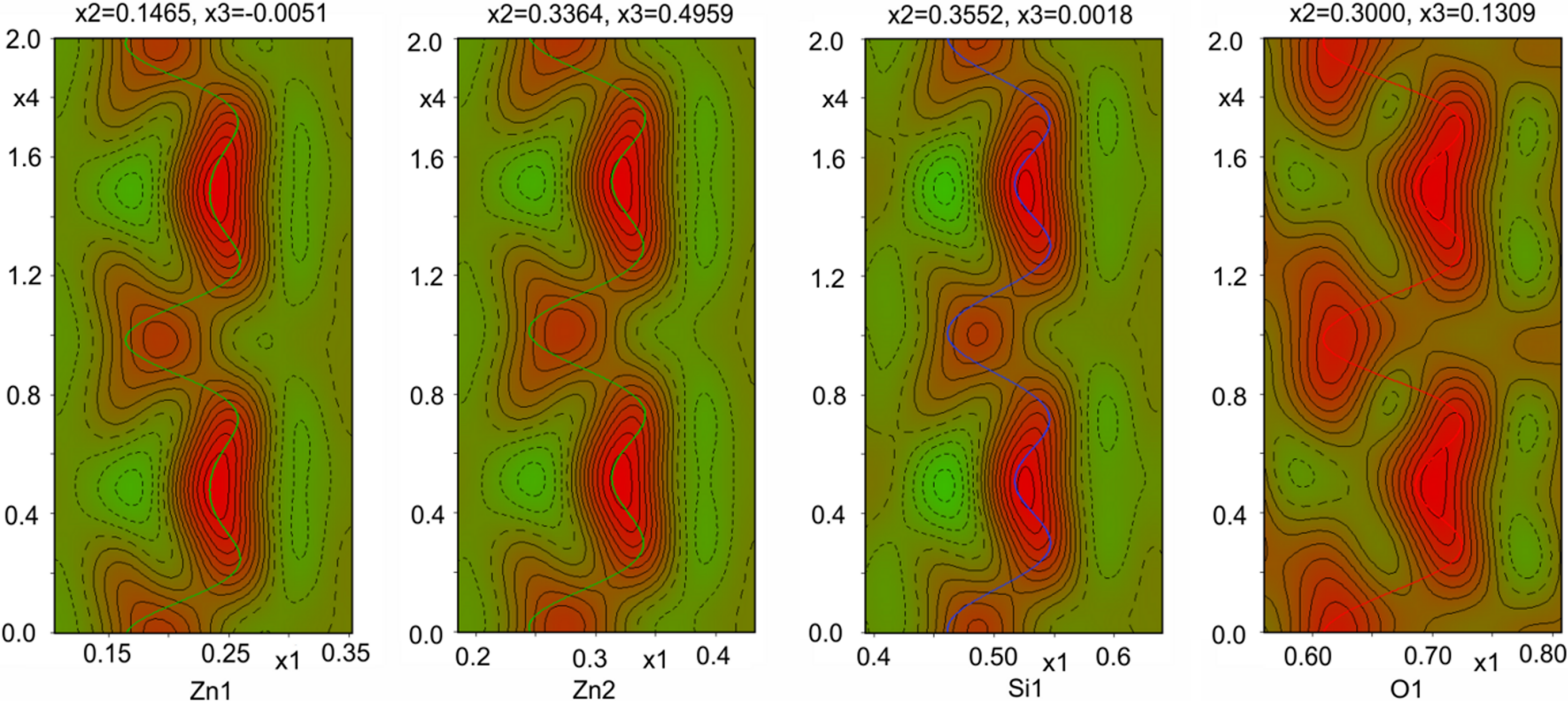
Fourier map, de Wolff’s section corresponding to the positions of atoms Zn1, Zn2, Si1 and O1 at 4.1 GPa. In this 2D contour plot *x*1 corresponds to the *X* axis and *x*4 is the phase of modulation. The contour values are as follows: 0.3 for Zn1 and Zn2, 1.5 for Si1, and 0.5 for O1. Dashed lines (green region) depict negative contours and the red region corresponds to the positive contours. Solid coloured lines (green, blue and red) represent the refined modulation function for zinc, silicon and oxygen atoms, respectively.

We solved and refined the structure of the high-pressure phase in the (3+1)D dimensional space group *Pnn*2(0, β, 0)000 using *Jana2020* (Petricek *et al.*, 2023 ▸), taking into account the satellite reflections up to the second order. The structure refinement revealed that the atoms change their position mostly along the *X*(*a*) axis. Relatively significant position modulations are observed for Zn1, Zn2, Si1 and O2 (see Fig. 9 ▸). The atoms fluctuate/jump between two main positions.

The 2D sections in Fig. 9 ▸ illustrate that the electron density contains reliable information about modulation, including variability in the positions of atoms. This type of map is obtained with *Jana2020* (Petricek *et al.*, 2023 ▸) and is called a de Wolff’s section (de Wolff, 1974 ▸). Each independent atom in the average structure is modulated by the application of a modulation function. As shown in the sections of the Fourier maps (Fig. 9 ▸), they exhibit a sinusoidal behaviour. The shape of this modulation suggests a continuous character. The refined model shows that the amplitudes of the displacive modulation have the main component along the *X*(*a*) axis. A summary of the refined amplitude displacements for zinc, silicon and oxygen atoms is presented in Tables 3 ▸–5 ▸ ▸.

As stated above, because the length of the modulation vector varies between 0.105 b* and 0.179 b*, to observe the whole modulation wave, one needs to present between five and nine unit cells, depending on the precise value of the modulation vector. To visualize what it means when atoms change their positions along the *X* axis, we present a so-called approximate structure in Figs. 9 ▸ and 10 ▸. This is no longer the averaged structure like in Fig. 1 ▸, where on the basis of one unit cell the whole atomic configuration is described. This time there are eight neighbouring unit cells reorganizing one by one along the *Y* axis.

**Figure 10:**
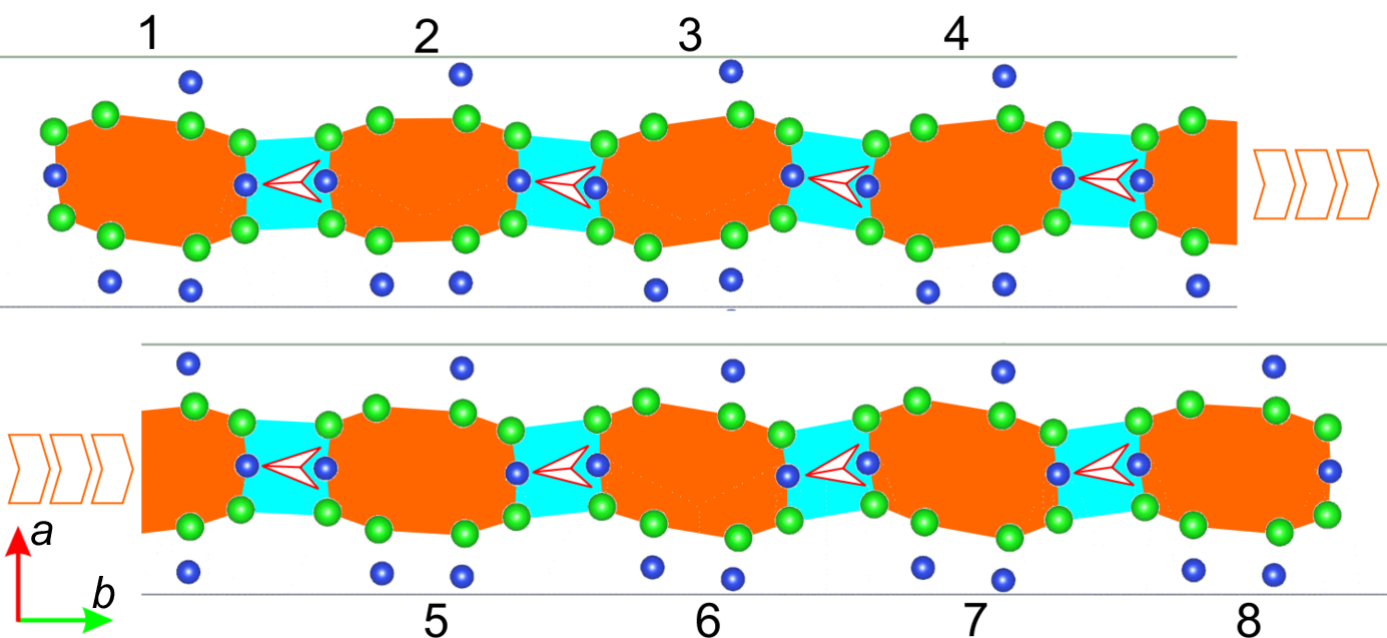
Modulation wave at 4.1 GPa. The modulation is along the [010] direction. Atoms change positions along the [100] direction. The green circles represent the positions of zinc atoms and the blue circles represent the positions of the silicon atoms. The oxygen and hydrogen atoms are omitted for clarity. The area between zinc atoms within a particular unit cell forms an irregular octagon, shown in orange.

In Fig. 10 ▸ (view of the *XY* plane along the [001] direction), the nature of the modulation is shown in a simplified and schematic way. One can easily observe how the positions of the zinc atoms (and shapes of particular octagons) change between neighbouring unit cells. The additional red arrow indicates how the orientation between two Si atoms within such a quadrangle changes; spaces between these orange octagons (quadrangles containing Zn and Si atoms) are coloured light blue. We simply see this modulation as a wave which goes across approximately eight neighbouring unit cells.

**Figure 11:**
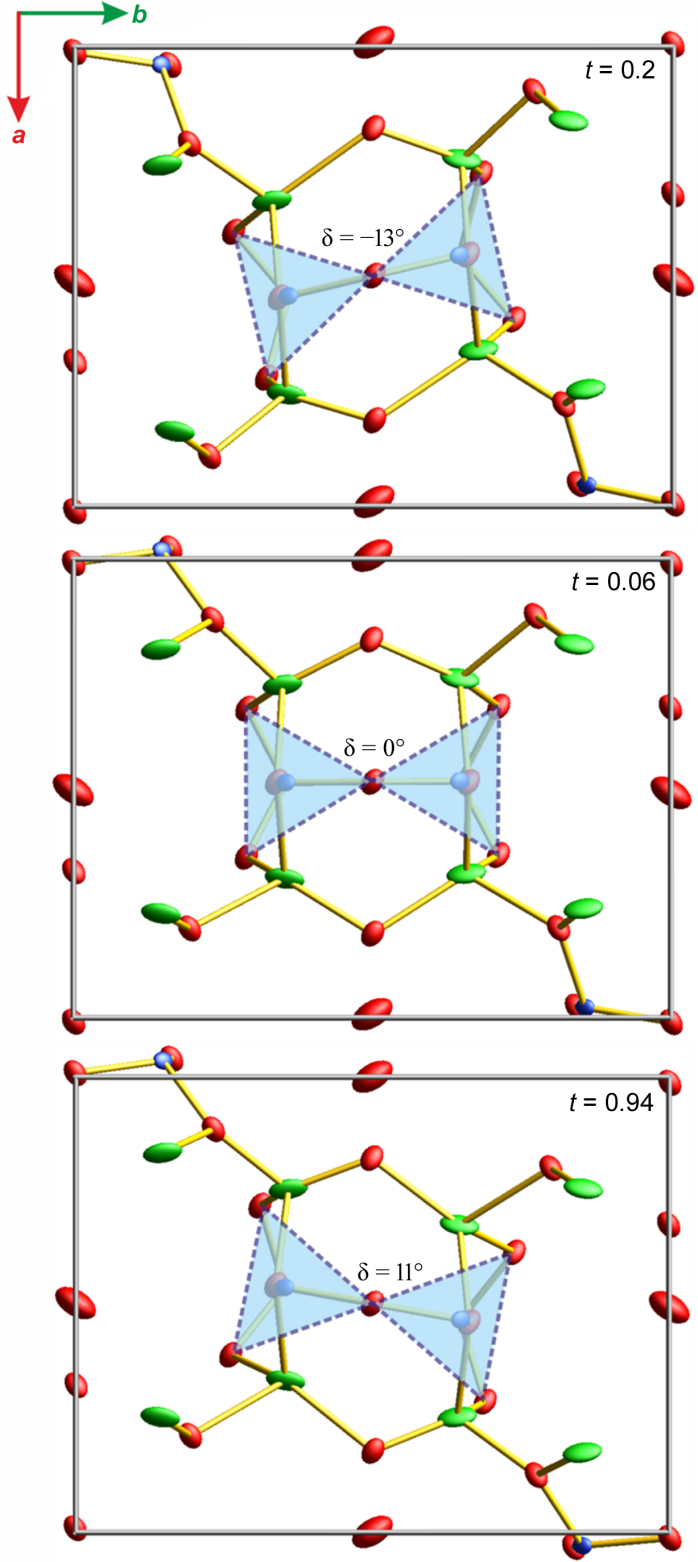
Modulation in the **Hmp** structure. Swinging of the SiO_4_ groups relative to the [010] direction defined by the angle δ (pressure point 4.1 GPa).

Fig. 11 ▸ shows the unit cell view along the *z* axis for the selected phase of the modulation, *t*. The angle δ between the Si—Si bond and unit-cell axis *x* (in the *xy* plane) is shown. The rigid SiO_4_ tetraherdal unit changes its position with the amplitude of δ from −13 to +11°. In our solution we have treated the SiO_4_ group as a rigid body.

**Figure 12:**
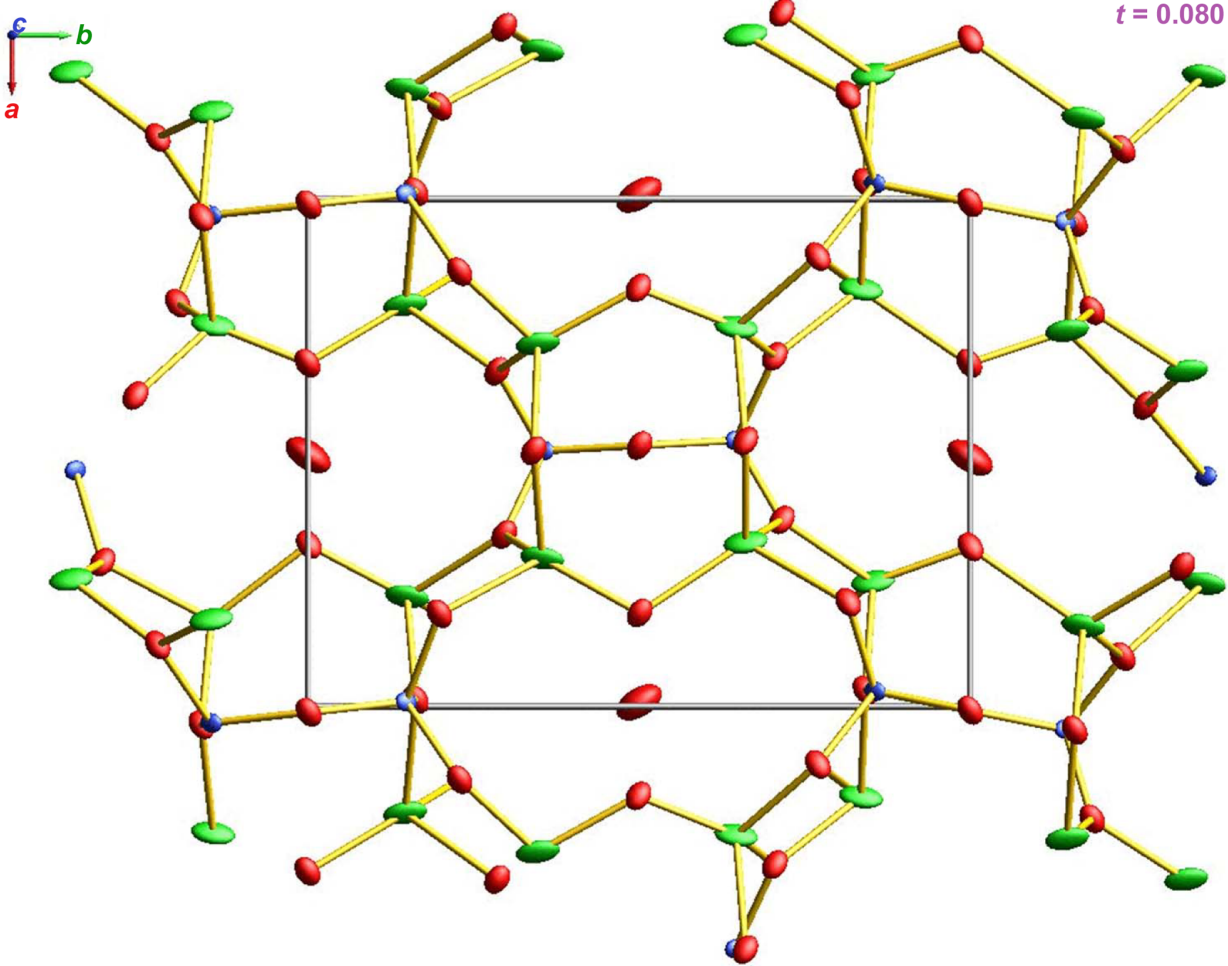
Atoms change positions as a consequence of displacive modulation. An animated version of the figure as a gif is included in the supporting information.

The positions and orientations of particular tetrahedra change for the neighbouring unit cells. In theory, the superposition of the unit cells depicted should provide us with the averaged structure as is shown in Fig. 12 ▸. An animated gif that shows how the atoms move within the unit cell due to modulation is given in the supporting information.

## 3. Conclusions

Single-crystal X-ray diffraction measurements conducted on hemimorphite under pressure confirm the phase transition from *Imm*2 to *Pnn*2. However, at a high pressure the structure is incommensurately modulated. The modulation vector lengths are slightly different for different sample crystals measured under the same pressure conditions but within the range from 0.105 b* to 0.179 b*. The first-order satellite reflections are observed in all cases, along the [010] direction. In one case, even the second-order satellites were observed and used for the structure refinement. Structure refinement which takes modulation into account was done in the space group *Pnn*2(0, β, 0)000. The mechanism of modulation involves changes in the positions of atoms mainly along the [100] direction. The atoms occupy two positions. The modulation amplitude increases at higher pressure. The first relatively weak satellite reflections appear at 2.3 GPa. At 2.6 GPa, the presence of satellite reflections is already very pronounced, suggesting that the structure previously reported in the literature at 2.44 GPa should be properly described in the space group *Pnn*2 (as the structure after the phase transition). The satellite reflections can be distinguished when the measurement is conducted with the use of a relatively intense X-ray beam and a good quality detector. In the case of poorer-quality data, it is possible to overlook the satellite reflections and solve the structure in a wrong space group. The use of a DAC causes restrictions of access to reciprocal space, and results in less complete data. Because different single crystals measured at the same pressure within the same DAC can show different lengths of the modulation vector, merging of the data is not possible, which makes accurate structure determination challenging.

## Supplementary Material

Crystal structure: contains datablock(s) 3_05GPa_Pnn2_DESY_crystal_2, 0_33GPa_Imm2_ESRF, 3_11GPa_Pnn2_DESY_crystal_2, 4_1GPa_Pnn2_DESY_crystal_3, 3_26GPa_Pnn2_DESY_crystal_1, 2_49GPa_Imm2_DESY_crystal_2, 3_50GPa_Pnn2_DESY_crystal_1, 3_19GPa_Pnn2_DESY_crystal_3, 2_8GPA_Pnn2_DESY_crystal_3, 1_92GPa_Imm2_DESY_crystal_3, 1_5GPa_Imm2_ESRF, global, I, 2_33GPa_Pnn2_ESRF, 3_3GPa_Pnn2_ESRF, 4_1GPa_Pnn2_ESRF, 1_0GPa_Imm2_ESRF, 2_1GPa_Imm2_ESRF, 2_6GPa_Pnn2_ESRF. DOI: 10.1107/S2052252524011060/fc5081sup1.cif

The nature of of the incommensurately modulated structure. DOI: 10.1107/S2052252524011060/fc5081sup2.gif

Supporting tables and figure. DOI: 10.1107/S2052252524011060/fc5081sup3.pdf

CCDC references: 2378030, 2378031, 2378032, 2378033, 2378034, 2378035, 2378036, 2378037, 2378038, 2378039, 2378040, 2378041, 2378042, 2378043, 2378044, 2378045, 2378046

## Figures and Tables

**Table 1:** Basic refinement parameters and data collection details for measurements at the ID27 (ESRF) beamline Structures after phase transition to space group *Pnn*2.

	2.3 GPa	2.6 GPa	3.3 GPa	4.1 GPa
*M*_r_	481.69	481.69	481.69	481.69
*a* (Å)	8.2199(6)	8.2161(2)	8.1356(8)	8.0899(13)
*b* (Å)	10.6921(2)	10.673(3)	10.6329(3)	10.5425(5)
*c* (Å)	5.0603(1)	5.0579(1)	5.0386(1)	5.0237(1)
*V* (Å^3^)	444.74(3)	443.54(13)	435.86(5)	428.46(7)
*F*(000)	464	464	464	464
*D_x_* (Mg m^−3^)	3.597	3.607	3.670	3.734
μ (mm^−1^)	0.46	0.46	0.47	0.48
Measured reflections	5555	10608†	4410	5096
Independent reflections	3878	5952	3231	3654
Observed [*I* > 2σ(*I*)] reflections	2167	3345	2475	2601
*R*_int_	0.018	0.022	0.016	0.019
θ (°)	θ_max_ = 19.9, θ_min_ = 1.6	θ_max_ = 19.9, θ_min_ = 1.6	θ_max_ = 19.9, θ_min_ = 1.6	θ_max_ = 20.0, θ_min_ = 1.6
(sin θ/λ)_max_ (Å^−1^)	1.524	1.526	1.528	1.537
Range of *h*, *k*, *l*	*h* = −13 → 10 *k* = −31 → 29 *l* = −15 → 15	*h* = −23 → 23 *k* = −31 → 29 *l* = −15 → 15	*h* = −13 → 10 *k* = −30 → 29 *l* = −15 → 15	*h* = −10 → 13 *k* = −29 → 31 *l* = −15 → 15
*R*[*F*^2^ > 2σ(*F*^2^)], *wR*(*F*^2^), *S*	0.033, 0.124, 1.00	0.056, 0.196, 1.08	0.063, 0.210, 1.20	0.198, 0.562, 2.43
No. of reflections	3878	5952	3231	3654
No. of parameters	74	82	74	66
(Δ/σ)_max_	0.001	0.021	0.001	0.001
Highest peak, deepest hole (e Å^−3^)	1.21, −0.88	5.40, −1.83	3.28, −3.13	20.59, −7.77

† Data are merged for two separate single crystals.

**Table 2:** Bond lengths and angles of the average structures of **Hmp** in the space group *Pnn*2

	2.3 GPa (this study)	2.6 GPa (this study)	3.01 GPa (Okamoto *et al.*, 2021 ▸)	3.17 GPa (Seryotkin & Bakakin, 2011 ▸)	3.3 GPa (this study)	4.1 GPa (this study)
*M*—O distance (Å)						
Si1—O1	1.607(3)	1.614(3)	1.592(14)	1.604(5)	1.613(8)	1.61(2)
Si1—O2	1.609(3)	1.622(3)	1.601(16)	1.609(5)	1.596(7)	1.558(15)
Si1—O3	1.6096(15)	1.615(2)	1.614(11)	1.618(5)	1.611(4)	1.625(8)
Si1—O5	1.6210(8)	1.6164(13)	1.623(9)	1.617(3)	1.6130(19)	1.598(4)
Zn1—O1	1.9456(15)	1.949(2)	1.941(12)	1.965(4)	1.933(4)	1.938(10)
Zn1—O2	1.9335(17)	1.937(3)	1.93(2)	1.922(5)	1.930(5)	1.921(10)
Zn1—O3	1.967(3)	1.966(2)	1.969(9)	1.962(5)	1.969(7)	1.968(16)
Zn1—O4	1.910(2)	1.924(4)	1.900(18)	1.922(5)	1.932(9)	1.93(3)
Zn2—O1	1.9416(17)	1.935(2)	1.94(2)	1.936(5)	1.936(4)	1.937(8)
Zn2—O2	1.9537(16)	1.941(2)	1.935(12)	1.953(4)	1.949(5)	1.957(11)
Zn2—O3	1.971(3)	1.968(2)	1.962(9)	1.955(5)	1.951(7)	1.914(16)
Zn2—O4	1.914(2)	1.914(3)	1.912(17)	1.912(5)	1.918(11)	1.85(3)

*M*—O—*M* angle (°)						
Si1—O5—Si1	145.59(15)	144.9(2)	144.1(13)	144.6(4)	143.8(4)	144.2(7)
Zn1—O3—Zn2	120.72(7)	121.00(10)	120.3(5)	119.61(7)	121.00(16)	120.8(4)
Zn1—O4—Zn2	124.6(2)	122.8(2)	124.1(10)	123.1(3)	120.6(9)	123(2)
Si1—O1—Zn1	116.27(10)	115.58(13)	116.4(8)	116.91(12)	115.9(3)	115.7(7)
Si1—O1—Zn2	127.65(10)	127.71(15)	125.1(12)	123.1(3)	124.7(4)	124.2(10)
Si1—O3—Zn1	119.42(14)	119.17(13)	119.2(5)	118.7(3)	118.7(4)	118.9(9)
Si1—O3—Zn2	119.24(15)	119.28(13)	120.0(6)	119.9(3)	119.9(4)	120.0(9)
Si1—O2—Zn2	115.67(10)	115.57(15)	115.2(8)	113.5(3)	115.2(3)	113.4(5)
Zn1—O1—Zn2	114.75(10)	115.13(12)	115.1(9)	115.3(2)	115.7(3)	115.3(6)
Zn1—O2—Zn2	114.71(10)	115.14(13)	114.7(9)	113.7(2)	114.5(3)	113.7(8)

**Table 3:** Experimental and crystallographic data measured on a single crystal at 4.1 GPa and refined with *Jana2020*

Crystal system, space group	Orthorhombic, *Pnn*2(0, β, 0)000
Temperature (K)	293
Pressure (kPa)	4100000
Wavevectors	**q** = 0.152 b*
*a*, *b*, *c* (Å)	8.0977(6), 10.5415(10), 5.0266(2)
*V* (Å^3^)	429.08(5)
*Z*	2
*F*(000)	456
*D_x_* (Mg m^−3^)	3.697
Radiation type	Synchrotron, λ = 0.2229 Å
No. of reflections	9580
θ range (°)	2.4–19.0
μ (mm^−1^)	0.47
Crystal shape	Plate
Colour	Colourless
Crystal size (mm)	0.05 × 0.03 × 0.02
Radiation source	Synchrotron
Detector resolution (pixels mm^−1^)	13.3333
Scan method	ω scans
Absorption correction	Multi-scan
*T*_min_, *T*_max_	0.545, 1
No. of measured, independent and observed [*I* > 2σ(*I*)] reflections	7097, 3955, 2127
*R*_int_	0.107
θ values (°)	θ_max_ = 10.7, θ_min_ = 1.5
(sin θ/λ)_max_ (Å^−1^)	0.834
Range of *h*, *k*, *l*	*h* = −13 → 13, *k* = −9 → 7, *l* = −8 → 8
Refinement on *F*	
*R*[*F*^2^ > 2σ(*F*^2^)], *wR*(*F*^2^),	All reflections: 0.188, 0.239; 1st sat. 0.194, 0.233; 2nd sat. 0.438, 0.548
*S*	7.72
No. of reflections	3955
No. of parameters	71
No. of restraints	3
No. of constraints	1
(Δ/σ)_max_	0.032
Highest peak, deepest hole (e Å^−3^)	4.80, −3.29

**Table 4:** Atomic positions

Atom	*x*	*y*	*z*	*U*_iso_
Zn1	0.2288(2)	0.1465(3)	−0.0051(3)	0.0251(9)
Zn2	0.3088(2)	0.3364(4)	0.4959(4)	0.0251(9)
Si1	0.5163(4)	0.3555(5)	0.0026(10)	0.010(2)
O1	0.6838(12)	0.300(2)	0.1309(19)	0.017(4)
O2	0.3625(13)	0.2815(17)	0.1337(18)	0.017(4)
O3	0.5162(15)	0.341(2)	−0.3171(8)	0.017(4)
O4	0.8186(10)	0.501(2)	0.5423(14)	0.017(4)
O5	0.5	0.5	0.101(2)	0.017(4)
O6	0.5	0	0.505(7)	0.05(2)

**Table 5:** Refined anisotropic displacement parameters

	U11	U22	U33	U12	U13	U23
Zn1, Zn2	0.0142(5)	0.053(3)	0.0084(4)	−0.0063(6)	−0.0049(5)	−0.0010(10)
Si1	0.0108(12)	0.013(7)	0.0071(8)	−0.0008(19)	0.0008(13)	0.005(2)
O1, O2, O3, O4, O5	0.023(2)	0.018(12)	0.0114(15)	−0.006(3)	−0.0049(13)	0.001(2)
O6	0.036(6)	0.06(6)	0.051(10)	0.021(12)	0	0

## Data Availability

The CIF may be obtained from FIZ Karlsruhe, 76344 Eggenstein-Leopoldshafen, Germany [fax: (+49)7247–808-666; e-mail: crysdata@fiz-karlsruhe.de] on quoting the CCDC deposition Nos. 2378030–2378046.
