# Supplementary figures and images for: Incommensurately modulated structure of Zn4Si2O7(OH)2·H2O at high pressure
Source: IUCrJ. 2025 Jan 1;12(Pt 1):62–73. doi: 10.1107/S2052252524011060 (PMC11707689; doi:10.1107/S2052252524011060)

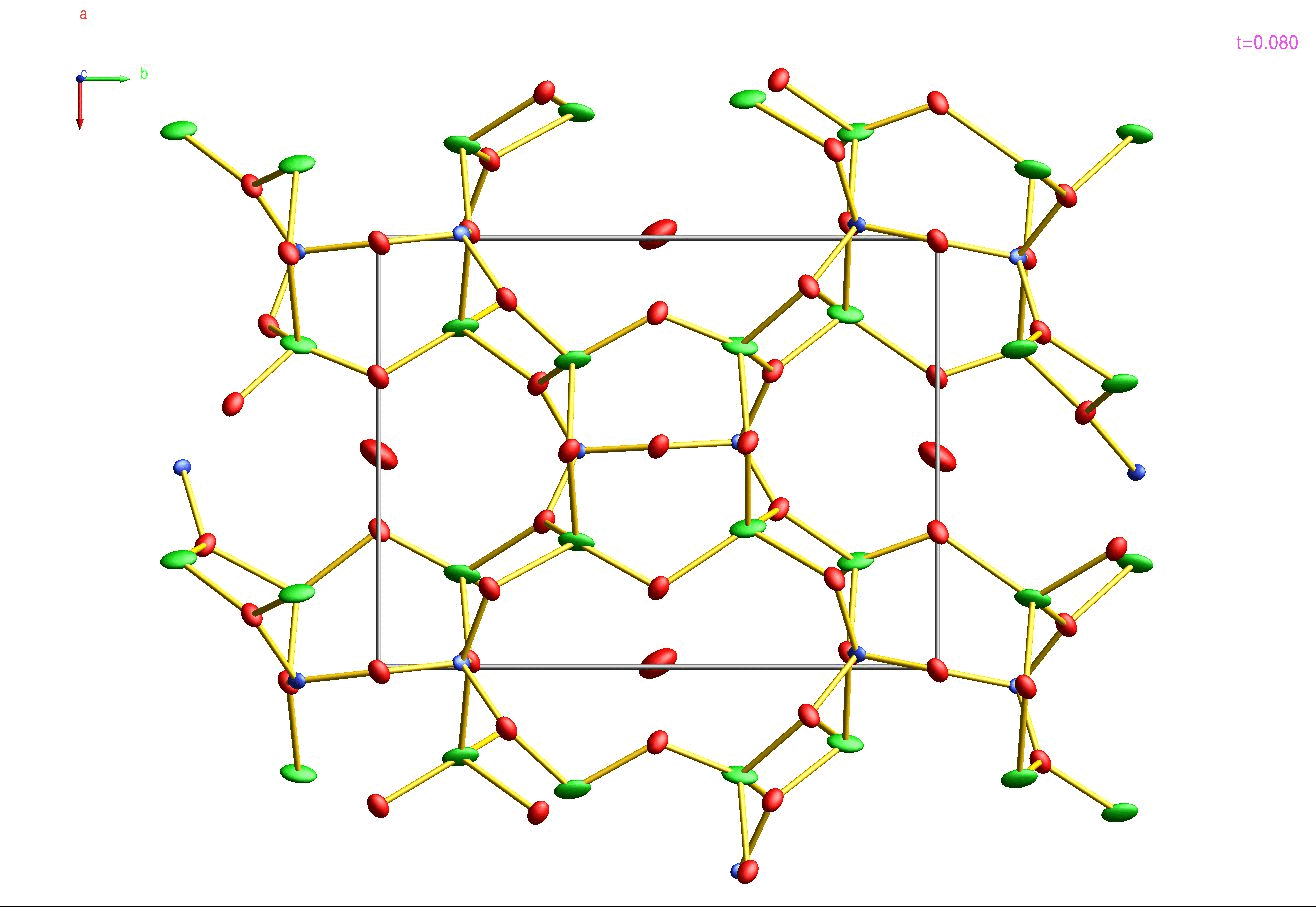

Supplement: Supplementary file 2 [file m-12-00062-sup2.gif]
