# Supplementary material for: Incommensurately modulated structure of Zn4Si2O7(OH)2·H2O at high pressure
Source: IUCrJ. 2025 Jan 1;12(Pt 1):62–73. doi: 10.1107/S2052252524011060 (PMC11707689; doi:10.1107/S2052252524011060)
Supplement: Supplementary file 3 [file m-12-00062-sup3.pdf]

IUCrJ

Volume 12 (2025)

Supporting information for article:

Incommensurately modulated structure of $Zn_4Si_2O_7(OH)_2 \cdot H_2O$ at high-pressure

Roman Gajda, Wojciech Sławiński, Tomasz Poręba, Jan Parafiniuk, Mohamed Mezouar, Przemysław Dera and Krzysztof Woźniak

Table S1 Independent atom model refinement basic parameters and data collection details collected at the ID27 (ESRF) beamline. Structures before phase transition to space group *Imm*2.

**Pressure (GPa)**	0.33	0.99	1.50	2.1
M_r	481.69	481.69	481.69	481.69
a **(Å)**	8.35707 (12),	8.31709 (17),	8.2846 (2),	8.2502 (2),
b **(Å)**	10.714 (2),	10.706 (3),	10.697 (2),	10.685 (3),
c **(Å)**	5.11006 (5)	5.09489 (7)	5.0823 (1)	5.0695 (1)
V **(Å^3)**	457.55 (9)	453.66 (12)	450.40 (10)	446.89 (11)
F**(000)**	464	464	464	464
D_x **(Mg m^{-3})**	3.496	3.526	3.552	3.580
**Radiation type**	λ = 0.2229 Å	λ = 0.2229 Å	λ = 0.2229 Å	λ = 0.2229 Å
**μ (mm^{-1})**	0.45	0.45	0.45	0.45
**Measured reflections**	7666	7678	7797	7661
**Independent reflections**	3085	3585	3625	3598
**Observed [$I > 2\sigma(I)$] reflections**	2451	2967	2922	2878
R_{int}	0.026	0.037	0.027	0.025
**θ values (°)**	θ_{max} = 20.2, θ_{min} = 1.5	θ_{max} = 20.2, θ_{min} = 1.5	θ_{max} = 19.9, θ_{min} = 1.5	θ_{max} = 20.4, θ_{min} = 1.5
**$(\sin \theta/\lambda)_{max}$ (Å^{-1})**	1.550	1.549	1.525	1.566
**Range of *h*, *k*, *l***	h = -24→23, k = -28→29, l = -14→15	h = -23→23, k = -29→31, l = -15→15	h = -23→23, k = -31→29, l = -15→15	h = -23→23, k = -31→30, l = -15→15
$R[F^2 > 2\sigma(F^2)]$, $wR(F^2)$, S	0.057, 0.158, 1.02	0.057, 0.170, 1.08	0.059, 0.171, 1.05	0.055, 0.162, 1.05
**No. of reflections**	3085	3585	3625	3598
**No. of parameters**	45	45	45	45
$(\Delta/\sigma)_{max}$	0.001	0.001	0.001	0.001
**Highest peak, deepest hole (e Å^{-3})**	6.19, -1.06	6.19, -3.35	6.23, -3.10	5.24, -2.12

At each pressure point, datasets from three single crystals were merged.

Table S2 Independent atom model refinement basic parameters and data collection details collected at the P24 (PetraIII, DESY) beamline (crystal #1).

**Pressure (GPa)**	3.26	3.50
**Space group**	*Pnn*2	*Pnn*2
M_r	477.66	477.66
a **(Å)**	8.1701 (4),	8.1412 (9),
b **(Å)**	10.6651 (3),	10.6227 (8),
c **(Å)**	5.0554 (1)	5.0448 (2)
V (Å^3)	440.50 (3)	436.28 (6)
F**(000)**	456	456
D_x (Mg m^{-3})	3.601	3.636
**Radiation type**	λ = 0.35424 Å	λ = 0.35424 Å
μ (mm^{-1})	1.64	1.66
**Measured reflections**	4747	2456
**Independent reflections**	1836	1841
**Observed** [$I > 2\sigma(I)$] **reflections**	1764	1654
R_{int}	0.023	0.033
**θ values (°)**	θ_{max} = 22.9, θ_{min} = 2.3	θ_{max} = 22.9, θ_{min} = 2.3
$(\sin \theta/\lambda)_{max}$ (Å^{-1})	1.096	1.099
**Range of** ***h*****,** ***k*****,** ***l***	h = -11→13, k = -18→19, l = -10→10	h = -13→11, k = -19→18, l = -10→10
$R[F^2 > 2\sigma(F^2)]$, $wR(F^2)$, S	0.028, 0.079, 1.12	0.045, 0.116, 0.98
**No. of reflections**	1836	1841
**No. of parameters**	74	74
$(\Delta/\sigma)_{max}$	0.001	0.001
**Highest peak, deepest hole (e Å**$^{-3}$**)**	0.74, -1.59	0.98, -1.56

Table S3 Independent atom model refinement basic parameters and data collection details collected at the P24 (PetraIII, DESY) beamline (crystal #2).

**Pressure (GPa)**	2.49	3.05	3.11
**Space group**	*Imm*2	*Pnn*2	*Pnn*2
M_r	477.66	477.66	477.66
a **(Å)**	8.2334 (7),	8.1695 (4),	8.1572 (3),
b **(Å)**	10.717 (6),	10.658 (3),	10.640 (3),
c **(Å)**	5.0641 (3)	5.0531 (1)	5.0499 (1)
V **(Å^3)**	446.8 (3)	439.99 (11)	438.31 (11)
F**(000)**	456	456	456
D_x **(Mg m^{-3})**	3.550	3.605	3.619
**Radiation type**	λ = 0.35424 Å	λ = 0.35424 Å	λ = 0.35424 Å
**μ (mm^{-1})**	1.62	1.64	1.65
**Measured reflections**	1340	4934	4480
**Independent reflections**	677	1289	1164
**Observed [$I > 2\sigma(I)$] reflections**	528	1183	1064
R_{int}	0.062	0.042	0.038
**θ values (°)**	θ_{max} = 23.3, θ_{min} = 2.2	θ_{max} = 23.0, θ_{min} = 2.2	θ_{max} = 22.9, θ_{min} = 2.2
**$(\sin \theta/\lambda)_{max}$ (Å^{-1})**	1.118	1.105	1.097
**Range of *h*, *k*, *l***	*h* = -14→13, *k* = -8→6, *l* = -8→8	*h* = -14→13, *k* = -7→8, *l* = -8→8	*h* = -14→13, *k* = -8→7, *l* = -8→8
$R[F^2 > 2\sigma(F^2)]$, $wR(F^2)$, S	0.050, 0.160, 1.03	0.049, 0.178, 1.19	0.039, 0.102, 1.04
**No. of reflections**	677	1289	1164
**No. of parameters**	42	59	74
$(\Delta/\sigma)_{max}$	0.001	0.001	0.001
**Highest peak, deepest hole (e Å^{-3})**	1.01, -1.59	0.96, -1.83	0.58, -0.86

Table S4 Independent atom model refinement basic parameters and data collection details collected at the P24 (PetraIII, DESY) beamline (crystal #3).

**Pressure (GPa)**	1.92	2.80	3.19	4.10
**Space group**	*Imm*2	*Pnn*2	*Pnn*2	*Pnn*2
M_r	477.66	477.66	477.66	477.66
a **(Å)**	8.2501 (2),	8.1865 (4),	8.1559 (2),	8.0991 (3),
b **(Å)**	10.694 (3),	10.670 (5),	10.636 (3),	10.540 (5),
c **(Å)**	5.06794 (16)	5.0479 (2)	5.0475 (1)	5.0319 (3)
V **(Å^3)**	447.12 (12)	440.93 (19)	437.85 (11)	429.5 (2)
F**(000)**	456	456	456	456
D_x **(Mg m^{-3})**	3.548	3.598	3.623	3.693
**Radiation type**	λ = 0.35424 Å	λ = 0.35424	λ = 0.35424 Å	λ = 0.35424 Å
**μ (mm^{-1})**	1.62	1.64	1.65	1.68
**Measured reflections**	2462	2790	5254	4899
**Independent reflections**	737	758	1406	1265
**Observed [$I > 2\sigma(I)$] reflections**	678	671	1281	1055
R_{int}	0.123	0.046	0.038	0.070
**θ values (°)**	θ_{max} = 23.6, θ_{min} = 2.4	θ_{max} = 22.9, θ_{min} = 1.6	θ_{max} = 22.8, θ_{min} = 1.6	θ_{max} = 22.5, θ_{min} = 2.4
**$(\sin \theta/\lambda)_{max}$ (Å^{-1})**	1.128	1.097	1.095	1.082
**Range of *h*, *k*, *l***	h = -16→16, k = -7→7, l = -10→9	h = -16→16, k = -7→7, l = -9→9	h = -16→16, k = -7→7, l = -9→9	h = -16→16, k = -6→6, l = -9→9
$R[F^2 > 2\sigma(F^2)]$, $wR(F^2)$, S	0.099, 0.252, 1.03	0.045, 0.130, 1.05	0.034, 0.096, 1.04	0.041, 0.115, 1.00
**No. of reflections**	737	758	1406	1265
**No. of parameters**	43	58	74	68
$(\Delta/\sigma)_{max}$	0.001	0.241	0.001	0.001
**Highest peak, deepest hole (e Å^{-3})**	2.49, -2.18	0.84, -0.91	0.75, -0.70	1.22, -0.89

Table S5 Independent atom model refinement basic parameters and data collection details collected at the XPress (Elettra) beamline.

**Pressure (GPa)**	1.11	2.57	3.03	3.15
**Space group**	*Imm*2	*Pnn*2	*Pnn*2	*Pnn*2
M_r	477.66	477.66	477.66	477.66
a **(Å)**	8.268 (8),	8.1874 (8),	8.1543 (14),	8.130 (8),
b **(Å)**	10.7329 (18),	10.738 (11),	10.665 (17),	10.6651 (16),
c **(Å)**	5.0951 (6)	5.0511 (3)	5.0462 (5)	5.0629 (5)
V **(Å^3)**	452.1 (4)	444.1 (5)	438.8 (7)	439.0 (4)
F**(000)**	456	456	456	456
D_x **(Mg m^{-3})**	3.509	3.572	3.615	3.613
**Radiation type**	λ = 0.4956 Å	λ = 0.4956 Å	λ = 0.4956 Å	λ = 0.4956 Å
**μ (mm^{-1})**	3.96	4.03	4.08	4.08
**Measured reflections**	477	671	826	651
**Independent reflections**	382	421	518	533
**Observed [$I > 2\sigma(I)$] reflections**	369	320	486	505
R_{int}	0.065	0.023	0.041	0.077
**θ values (°)**	θ_{max} = 26.2, θ_{min} = 4.2	θ_{max} = 25.8, θ_{min} = 3.1	θ_{max} = 25.3, θ_{min} = 3.6	θ_{max} = 24.4, θ_{min} = 2.7
**$(\sin\theta/\lambda)_{max}$ (Å^{-1})**	0.890	0.878	0.861	0.834
**Range of *h*, *k*, *l***	$h = -6\rightarrow5$, $k = -17\rightarrow17$, $l = -9\rightarrow8$	$h = -12\rightarrow12$, $k = -5\rightarrow4$, $l = -8\rightarrow8$	$h = -12\rightarrow12$, $k = -5\rightarrow4$, $l = -8\rightarrow8$	$h = -4\rightarrow5$, $k = -16\rightarrow16$, $l = -8\rightarrow8$
**$R[F^2 > 2\sigma(F^2)]$, $wR(F^2)$, S**	0.100, 0.261, 1.26	0.172, 0.446, 2.13	0.163, 0.449, 2.38	0.114, 0.293, 1.42
**No. of reflections**	382	421	518	533
**No. of parameters**	30	43	48	48
$(\Delta/\sigma)_{max}$	0.001	0.001	0.001	0.001
**Highest peak, deepest hole (e Å^{-3})**	2.39, -2.51	3.62, -3.44	3.08, -4.10	2.07, -3.02

Table S6 Amplitude displacement for the sine and cosine terms of the first order of the harmonics in the Fourier series corresponding to the zinc, silicon and oxygen atoms.

atom	axis	cosine	sine
Zn1	x	-0.0287(3)	0.0035(3)
	y	0	0
	z	-0.0040(5)	0.0061(5)
Zn2	x	-0.0316(3)	-0.0026(3)
	y	0.0159(5)	0
	z	0.0023(5)	0.0056(5)
Si1	x	-0.0280(4)	-0.0013(6)
	y	-0.00182(5)	0.000253(7)
	z	0	0.0000690(18)
O1	x	-0.0384(7)	0.0020(6)
	y	-0.0203(3)	0.00392(6)
	z	0.0001484(10)	0.000768(5)
O2	x	-0.0418(7)	0.0034(6)
	y	0.0151(3)	-0.00320(6)
	z	-0.0001212(12)	-0.000573(6)
O3	x	-0.0306(6)	0.0000(6)
	y	-0.00178(17)	0.00027(3)
	z	0	0.000068(6)
O4	x	0.003(2)	-0.0051(14)
	y	0	0
	z	-0.004(3)	-0.0075(18)
O5	x	0	-0.0053(6)
	y	0	0
	z	0	0
O6	x	0	0.010(3)
	y	0	0
	z	0	0

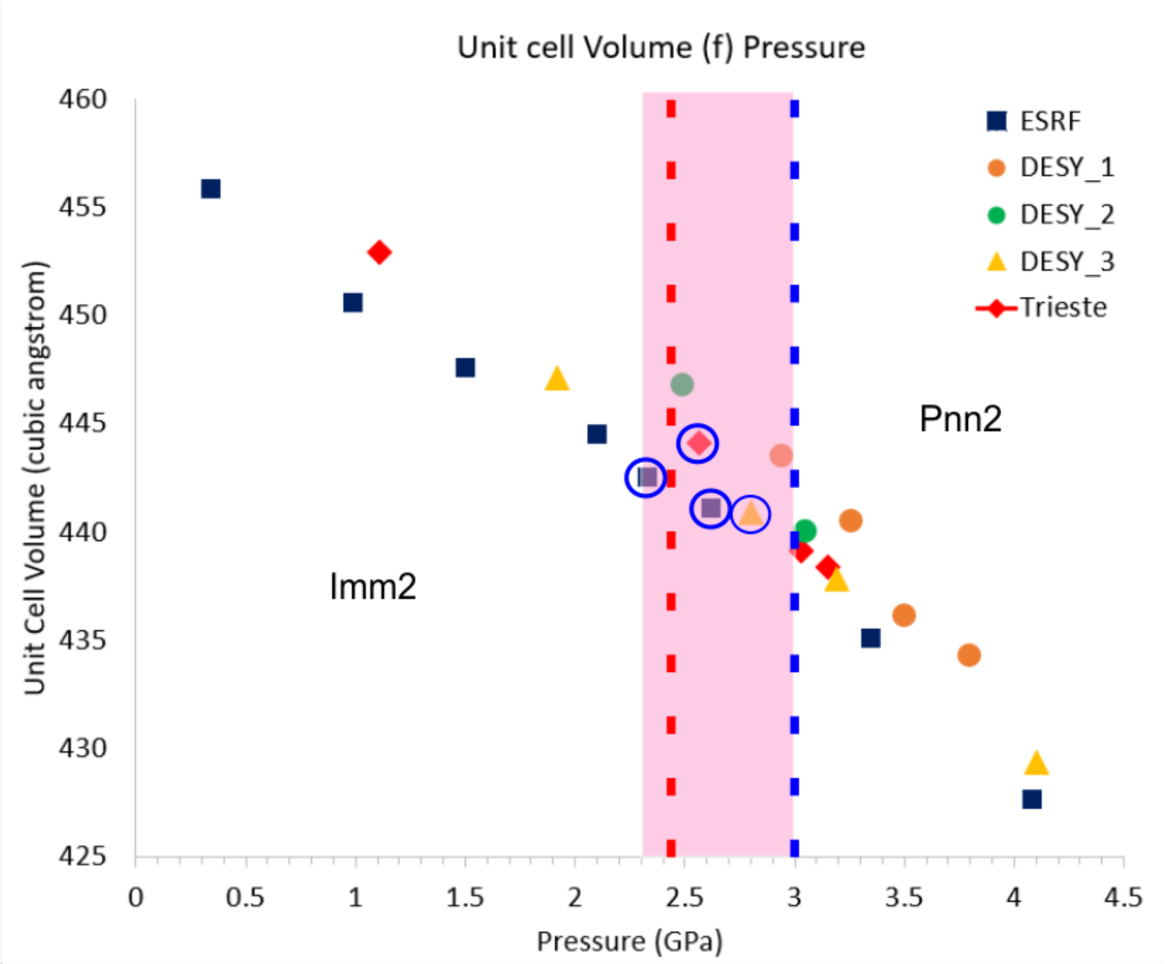

Figure S1 The unit cell volume as a function of pressure. Comparison of datasets collected at different synchrotron facilities.
